# Glycation of the Major Milk Allergen β‐Lactoglobulin Changes Its Allergenicity by Alterations in Cellular Uptake and Degradation

**DOI:** 10.1002/mnfr.201800341

**Published:** 2018-07-29

**Authors:** Marija Perusko, Manon van Roest, Dragana Stanic‐Vucinic, Peter J. Simons, Raymond H. H. Pieters, Tanja Cirkovic Velickovic, Joost J. Smit

**Affiliations:** ^1^ Faculty of Chemistry–Innovation Center d.o.o. 11000 Belgrade Serbia; ^2^ Institute for Risk Assessment Sciences Immunotoxicology Group Utrecht University 3584 CM Utrecht The Netherlands; ^3^ Center of Excellence for Molecular Food Sciences and Department of Biochemistry University of Belgrade–Faculty of Chemistry 11000 Belgrade Serbia; ^4^ Bioceros B.V. 3584 CM Utrecht The Netherlands; ^5^ Faculty of Bioscience Engineering Ghent University 9000 Ghent Belgium; ^6^ Ghent University Global Campus Yeonsu‐Gu 21985 Incheon South Korea

**Keywords:** β‐lactoglobulin, food allergens, food processing, Maillard reaction, uptake and degradation by DCs

## Abstract

**Scope:**

During food processing, the Maillard reaction (МR) may occur, resulting in the formation of glycated proteins. Glycated proteins are of particular importance in food allergies because glycation may influence interactions with the immune system. This study compared native and extensively glycated milk allergen β‐lactoglobulin (BLG), in their interactions with cells crucially involved in allergy.

**Methods and results:**

BLG was glycated in MR and characterized. Native and glycated BLG were tested in experiments of epithelial transport, uptake and degradation by DCs, T‐cell cytokine responses, and basophil cell degranulation using ELISA and flow cytometry. Glycation of BLG induced partial unfolding and reduced its intestinal epithelial transfer over a Caco‐2 monolayer. Uptake of glycated BLG by bone marrow–derived dendritic cells (BMDC) was increased, although both BLG forms entered BMDC via the same mechanism, receptor‐mediated endocytosis. Once inside the BMDC, glycated BLG was degraded faster, which might have led to observed lower cytokine production in BMDC/CD4^+^ T‐cells coculture. Finally, glycated BLG was less efficient in induction of degranulation of BLG‐specific IgE sensitized basophil cells.

**Conclusions:**

This study suggests that glycation of BLG by MR significantly alters its fate in processes involved in immunogenicity and allergenicity, pointing out the importance of food processing in food allergy.

## 1 Introduction

Nowadays, the majority of food is subjected to thermal treatment prior to consumption, which induces structural and chemical modifications of food proteins. One of the most widespread modifications is glycation by the Maillard reaction (MR), a very complex reaction cascade that occurs between amino and carbonyl groups.^1^ Products of MR are very diverse and give rise to a variety of glycation structures covalently attached to food proteins that are collectively termed as advanced glycation endproducts (AGEs). Glycated proteins possess many functional properties that are relevant for food industry such as improved gelling, emulsifying and foaming activity, increased temperature and pH stability, and increased antioxidant activity,^2^, ^3^, ^4^, ^5^, ^6^ but also characteristics that are attractive for consumers such as appealing color, smell, taste, and texture.^7^

Apart from improving techno‐functional properties, MR also alters biological and immunological properties of food proteins. It is well documented that glycation of food allergens affects their IgE binding ability by direct masking of linear as well as conformational epitopes, leading to reduced allergenicity.^8^, ^9^ In contrast, other studies have pointed out that glycation led to formation of neoantigens, hereby increasing their recognition by IgE antibodies.^10^, ^11^, ^12^ Recently, evidences are emerging that effects of MR on food allergens go beyond IgE recognition. Dendritic cells (DCs), as the most important antigen‐presenting cells in the immune system, possess several receptors that recognize glycation structures, including receptor for advanced glycation end products (RAGE), scavenger receptors (SRs), galectin‐3, and CD‐36.^13^, ^14^ Acting through these receptors, glycation structures may influence recognition, uptake, and processing of food allergens by DCs. Subsequently, this may have consequences on the maturation of DCs and subsequent activation of naïve T‐cells. For instance, a glycated form of the major egg allergen, ovalbumin (OVA) binds to SR class A which mediates increased allergen uptake by DCs, and leads to a stronger T helper type 2 cytokine response.^15^, ^16^ Similarly, AGE‐BSA induced maturation of DCs and augmented their capacity to stimulate T‐cell proliferation and cytokine secretion.^17^ It has even been speculated that AGEs of food allergens interact with DCs, promote immune responses, and contribute to the development of food allergy by mimicking endogenous danger signals.^18^

Very little is known about allergen processing and degradation inside DCs, despite that this is of great importance. It has been described that the dynamics of uptake and degradation inside endolysosomal compartments of DCs affects class II MHC loading pathways, antigen presentation, and finally T‐cell polarization.^19^, ^20^, ^21^

β‐Lactoglobulin (BLG) is the most abundant whey protein and one of the major allergens in milk.^22^ Whey proteins are frequently used as food additives because of their valuable nutritional and techno‐functional properties. Thus, they are very likely to be glycated in MR upon food processing. There are scarce literature data about the effects of the MR on the immunogenic properties of the major whey allergen. Hence, the aim of this study was to glycate BLG with glucose to a high extent and to conduct a comparative analysis, relative to its native form, regarding their behavior in processes that are involved in immunogenicity and allergenicity. Intestinal epithelial transcytosis, uptake and degradation by BMDCs, ability to induce cytokine production by BLG‐specific CD4^+^ T‐cells, and basophil degranulation capacity of native and glycated BLG were investigated.

## 2 Experimental Section

### 2.1 Chemicals and Standards

Unless otherwise stated, all chemicals were obtained from Sigma‐Aldrich (St. Louis, MO, USA). For all experiments, Milli‐Q water (Millipore, France) was used.

### 2.2 Mice

Five‐week‐old female C3H/HeOuJ mice were purchased from Charles River (France) and were maintained in specific pathogen‐free conditions within the animal care facility at the Utrecht University. Experiments were approved by the Animal Experiments Committee of the Utrecht University.

### 2.3 Protein Sample Preparation

BLG was isolated from raw cow's milk as described previously^23^ (details are given in supporting information). Purity of the obtained BLG preparation was estimated to be >95% by SDS‐PAGE. Protein concentration was determined by bicinhoninic acid (BCA) assay (Pierce, Amsterdam, the Netherlands). Endotoxin was reduced from samples by affinity chromatography on ε‐poly‐l‐lysine cellulose beads (Thermo Scientific, Rockford, IL). The residual amount of endotoxin was determined using LAL Chromogenic Endotoxin Quantitation Kit (Thermo Scientific, Rockford, IL). The endotoxin concentration in BLG sample was 0.4 EU mg^−1^ of protein.

### 2.4 Glycation Procedure and Characterization of Glycated BLG

Glycated BLG was made by performing the MR in which 10 mg mL^−1^ BLG was incubated with 180 mg mL^−1^ glucose in 50 mm phosphate buffer, pH 8.0 at 60 ˚C for 10 days in capped vials. Thermally treated BLG under the same conditions but without glucose was also prepared. To determine the degree of glycation, free amino groups were estimated by the ortho‐phthalaldehyde (OPA) method as described previously.^24^ To further characterize glycated BLG, SDS‐PAGE was performed on 14% gel (5 μg of protein per lane, under reducing and nonreducing conditions. Protein bands were visualized by Coomassie Brilliant Blue staining. Structural changes were monitored by recording far‐UV CD spectra on a JASCO J‐815 spectropolarimeter (JASCO, Tokyo, Japan) of 0.85 mg mL^−1^ native and glycated BLG in 50 mm sodium phosphate buffer pH 8.0. Each spectrum was acquired four times, and the results were averaged. Spectra were analyzed by the CONTIN program to determine the proportion of secondary structures based on reference protein set of 29 soluble proteins—SP29 as previously was done.^23^

### 2.5 Caco‐2 Cell Culture

The culturing of Caco‐2 cells is described in supporting information.

### 2.6 Transport Across Caco‐2 Cell Monolayer

For transport studies, Caco‐2 cells were seeded at 1.6 × 10^4^ cells per insert on polycarbonate membranes with a pore size of 0.1 μm in 24‐well plates (Transwell, Corning Costar, Cambridge, MA). Cells at passages of 17–25 were used in experiments. Prior to the transport studies, transepithelial electrical resistance (TEER) was measured using Millicell‐ERS VoltOhmmeter (Millipore, Amsterdam, the Netherlands). Only cell monolayers with TEER >500 Ω were used. Native and glycated BLG were diluted in DMEM culture medium to a final concentration of 1.5 mg mL^−1^, and 100 μL of these preparations were loaded onto apical surface of Caco‐2 monolayer, while 600 μL of DMEM culture medium was added to the receiver compartment. Aliquots of 30 μL were withdrawn from the receiver compartment after 15, 30, 60, 120, 240, and 300 min and receiver compartment was replenished every time with medium. Transported native and glycated BLG were detected by commercially available ELISA kit (Bethyl Laboratories, Inc., Montgomery) according to manufacturer's instructions.

### 2.7 Generation of Bone Marrow–Derived Dendritic Cells (BMDCs)

Bone marrow cells isolated from femurs/tibias of naïve C3H/HeOuJ mice were cultured in conditioned‐complete DCs medium (RPMI 1640 medium glutaMAX [Gibco, Invitrogen, Carlsbad, USA]) supplemented with 10% heat‐inactivated FCS, 1 mm sodium pyruvate, 100 units mL^−1^ penicillin, 100 μg mL^−1^ streptomycin, 1% nonessential amino acids, 0.1 mm 2‐mercaptoethanol), with 10 ng mL^−1^ GM‐CSF (R&D, Oxon, UK) at 37 °C in a humidified atmosphere of 5% CO_2_. Medium was refreshed on day 3. Only 6 days old BMDCs were used for the following experiments.

### 2.8 Allergen Uptake by BMDCs

Native and glycated BLG were labeled with FITC according to the manufacturer's instructions (Sigma‐Aldrich, St. Louis, MO, USA). FITC‐native BLG and FITC‐glycated BLG conjugates were separated from unreacted FITC label using PD‐10 desalting column (GE Healthcare, Uppsala, Sweden). The labeling efficacy was the same for native and glycated BLG as determined by absorbance at 280 and 495 nm. The concentration of labeled samples was determined by BCA assay, and the samples were stored protected from light at −20 °C until use.

FITC‐native and FITC‐glycated BLG (10 μg mL^−1^) were incubated with 1 × 10^6^ BMDCs per mL of cell culture medium for 0, 5, 10, 20, 30, 45, 60, 90, and 120 min at 37 °C. Uptake was recorded using BD Accuri C6 Cytometer (BD Biosciences, San Jose, CA, USA). Mean fluorescence intensity (MFI) and percentage of FITC‐positive BMDCs were calculated using BD Accuri C6 software.

### 2.9 Mechanistic Studies of Allergen Uptake by BMDCs

To identify uptake mechanism for native and glycated BLG, BMDCs were pretreated with 10 μg mL^−1^ latrunculin B, 1 μm jasplakinolide, 2 mm amiloride, 2 μm phenylarsine oxide, inhibitors of phagocytosis, macropinocytosis, and receptor‐mediated endocytosis, respectively. To inhibit uptake mediated by specific receptors, BMDCs were incubated with the following inhibitors: 100 μg mL^−1^ dextran sulfate, or 200 μg mL^−1^ polyinosine specific for SRs, and 0.3 μm FPS‐ZM1 specific for RAGE. After 30 min of incubation at 37 °C with the above inhibitors, BMDCs were loaded with 10 μg mL^−1^ FITC‐native BLG and FITC‐glycated BLG. Uptake was recorded after 30 min of incubation at 37 °C by flow cytometry as described above.

### 2.10 Confocal Microscopy

1 × 10^5^ BMDCs in the presence or absence of different inhibitors under above described conditions were withdrawn, washed, and spun onto glass slides by cytospin. Cells were imaged on a Leica TCS SPE‐2 confocal laser scanning‐microscope on a DMI4000.

### 2.11 Endolysosomal Degradation Assay

Native and glycated BLG were covalently coupled to polystyrene beads (Polysciences, Inc., Warrington, PA, USA) (protocol included in supporting material).

Endolysosomal degradation was estimated according to Hoffmann et al.^25^ with minor modifications. Total of 12.5 × 10^6^ BMDCs from naïve mice were incubated with 50 × 10^6^ of native and glycated BLG‐coated beads at 37 °C for 15 min to allow phagocytosis. Phagocytosis was stopped by adding ice‐cold PBS. Non‐internalized beads were washed out three times with FCS floatation cushion at +4 °C. BMDCs were resuspended in 2.5 mL of conditioned‐complete DCs medium and incubated at 37 °C to start phagosome maturation. Aliquots of 480 μL were withdrawn after 0, 30, 120, 240, and 720 min and immediately mixed with 1 mL of ice‐cold PBS to stop phagosome maturation, and centrifuged for 5 min at 400 g at 4 °C. Pelleted BMDCs were lysed with lysis buffer (50 mm Tris, 150 mm NaCl, 0.5% Triton x, 1 mm DTT, 10 μg mL^−1^ Dnase I, pH 7.4, with the addition of complete protease inhibitor cocktail [Roche, Lewes, UK]), overnight, at 4 °C. Non‐degraded BLG on the beads surface was detected using rabbit polyclonal anti‐BLG antibody 1:1000 (Fitzgerald, 70R‐LR011) and anti‐rabbit Alexa 488 antibody (Invitrogen A11034). Alexa intensity was measured by flow cytometry.

### 2.12 Assessment of T‐cell Activation and Cytokine Production

BLG‐specific CD4^+^ T‐cells were obtained from spleens of C3H/HeOuJ mice immunized with BLG and aluminum hydroxide (Imject Alum, Pierce, the Netherlands). On days 0 and 7, three mice were intraperitoneally injected with 100 μg of native BLG and 1 mg of ALUM. On day 14, BMDCs from naïve mice were primed with 50 μg mL^−1^ of different forms of BLG for 2 h. BMDCs were scraped, washed, and transferred to a 48‐well plate where they were cocultured (1:10 cell number ratio) with freshly isolated CD4^+^ T‐cells for the next 72 h. Subsequently, levels of IFN‐γ, IL‐5, IL‐10, and IL‐13 were measured in coculture supernatants by commercially available ELISA (e‐Bioscience, Austria).

### 2.13 Anti‐BLG IgE Crosslinking‐Induced NFAT‐Responsive Luciferase in Basophilic RS‐ATL8 Cells

The humanized rat basophilic leukemia cell line RS‐ATL8 was kindly provided by R. Nakamura from the National Institute for Health Sciences, Tokyo, Japan. This cell line which was established by introducing the NFAT‐responsive luciferase gene into human FcεRI expressing RBL‐SX38 cells was used to measure IgE crosslinking‐induced luciferase expression (EXiLE). EXiLE assay was performed as described previously^26^ with slight modifications. RS‐ATL8 cells (5 × 10^4^ cells per 50 μL per well) in 96‐wells flat bottom culture plate were sensitized overnight with a pool of six anti‐BLG chimeric IgE monoclonal antibodies (1 μg mL^−1^ each individual humanized IgE).^27^ Cells were washed once with sterile PBS and then stimulated for 1 h at 37 °C in a 5% CO_2_ incubator with 50 μL per well of native and glycated BLG diluted in MEM containing 10% FCS in four different concentrations (1, 10, 100, and 1000 ng mL^−1^) or with 1 μg mL^−1^ goat anti‐human IgE antibodies (DakoCytomation, Glostrup, Denmark) as control. After stimulation, 50 μL of luciferase substrate solution containing cell lysis reagent (ONE‐Glo, Promega Corp., Tokyo, Japan) was added to the cells, and chemiluminescence was measured. Luciferase expression levels are represented as the fold increase of relative light units compared with the background expression.

### 2.14 Statistical Analysis

Data are presented as mean ± stand deviation (SD) or standard error of mean (SEM) and analyzed using GraphPad Prism software (La Jolla, CA). Data were tested by Student's *t*‐test. Differences were considered significant when *p*‐values were *<*0.05.

## 3 Results

### 3.1 BLG Was Glycated to a High Extent Accompanied by Modifications in Secondary Structure

**Figure 1:**
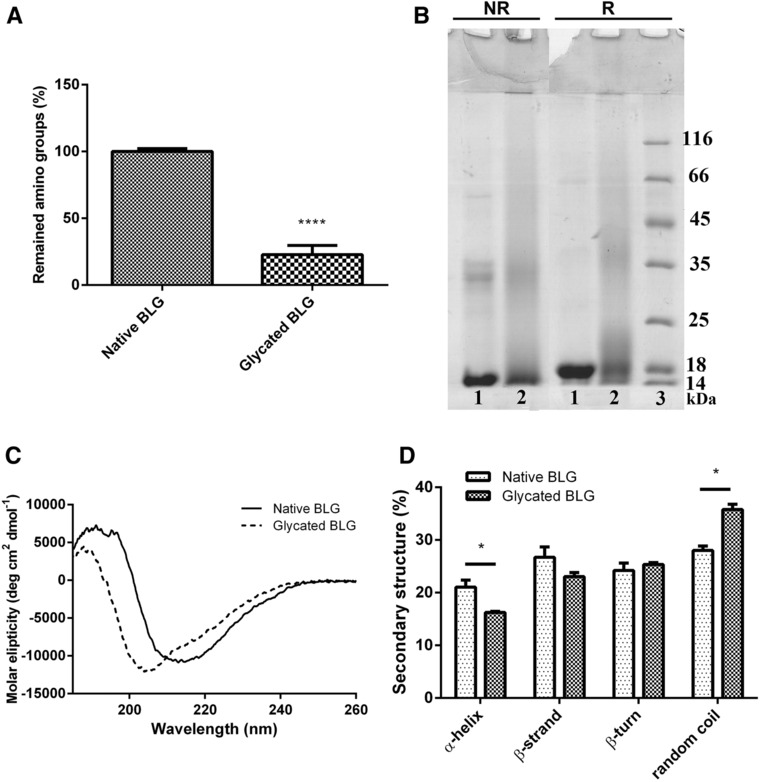
Characterization of BLG glycated in MR. A) Estimation of the degree of MR by determining remaining free amino group content by OPA method. Free amino group content was statistically compared by Student's *t*‐test. ****Denotes significance at *p* < 0.0001 confidence level. B) Protein profile of native and glycated BLG on 14% SDS‐PAGE gel under nonreducing (NR) and reducing (R) conditions. Protein bands were stained with CBB. 1) native BLG; 2) glycated BLG; 3) molecular weight markers (kDa). C) CD spectra of native and glycated BLG in far‐UV spectral range. Samples were recorded in 50 mm sodium phosphate buffer, pH 8.0 at protein concentration 0.85 mg mL. D) Percentages of the secondary structures were estimated by CONTIN algorithm available in CDPro package based on SP29 reference set. Secondary structure fractions were compared by Student's *t*‐test. *Represents significance at *p* < 0.05 confidence level.

BLG was glycated with glucose in the MR, and the degree of glycation was assessed by determining the free amino group content by OPA method (**Figure** 1A). A significant loss of available amino groups was observed; about 75% of amino groups were glycated.

Analysis of native and glycated BLG by SDS‐PAGE showed a band at 18 kDa, corresponding to BLG (Figure 1B). This band smeared toward higher molecular masses after conjugation with glucose molecules. Under nonreducing conditions, BLG dimers were present in both, native and glycated BLG, while under reducing conditions, native BLG was visible only in monomeric form, but glycated BLG was still visible in dimeric form, indicating the formation of covalent bonds other than disulfide bridges. Glycation reaction also induced covalent aggregates formation, noticeable as higher molecular masses at the interface of stacking and running gels.

Changes were observed in far‐UV CD spectra of glycated BLG compared to native BLG spectra (Figure 1C). The characteristic minimum of native BLG at 215 nm was shifted toward a lower wavelength, indicating a decrease in the ordered secondary structure. Software analysis of CD spectra showed a decrease in α‐helix accompanied with an increase in random coils (Figure 1D), suggesting a direct conversion of regular into irregular structures upon glycation.

In addition, thermal treatment of BLG without glucose led to different changes in structure and a higher extent of aggregate formation in comparison to glycated BLG, as evidenced by SDS‐PAGE under reducing and nonreducing conditions (Figure S1A,B, Supporting Information). Also, far‐UV CD spectra analysis showed differences in the structural change of heated and glycated BLG in comparison to native BLG. Heated BLG showed a significantly higher loss of β‐sheets (Figure S2A,B, Supporting Information). The protective effect of sugar on the heat‐induced denaturation and aggregation of BLG has been shown extensively before.^5^, ^24^, ^28^, ^29^ Therefore structural changes induced by Maillard reaction are not simply the sum of thermally induced structural changes and covalent attachment of sugar moieties. Thus, native BLG, rather than heated BLG, was used as control in order to compare their interactions with immune cells crucially involved in cow`s milk allergy.

### 3.2 Glycation of BLG Reduced Transport Across Caco‐2 Cell Monolayer

**Figure 2:**
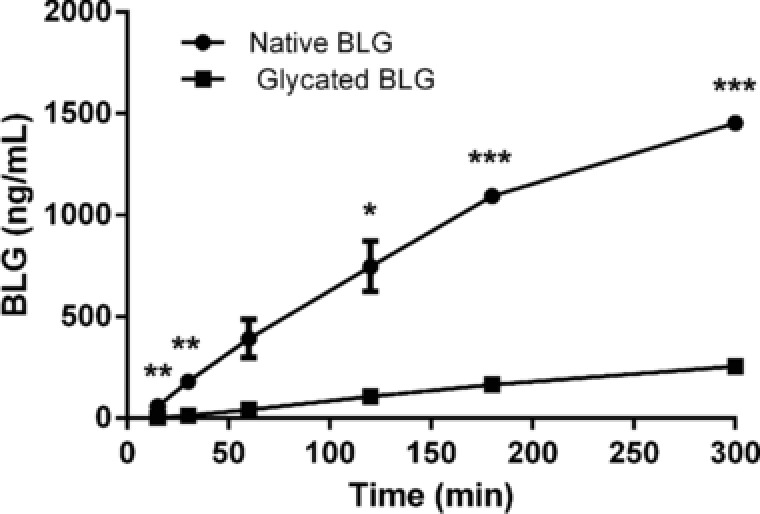
Bioavailability of native and glycated BLG in vitro. Transcytosis of native and glycated BLG across Caco‐2 monolayer. Transport was measured by determining BLG concentration in samples withdrawn from basolateral chamber at different time‐points up to 3 h. Each point represents mean ± SD. One representative experiment out of three independent is shown. *, **, and *** represent significance at *p* < 0.05, 0.01, and 0.001, respectively.

Transport of BLG samples across Caco‐2 cell monolayer, a model of intestinal barrier function, was examined. Native and glycated BLG were applied on apical side of Caco‐2 cells and transport was followed in time by determining BLG concentration on basal side. Glycation reaction dramatically reduced detection of BLG at basolateral side of Caco‐2 monolayer (**Figure** 2). Transport of both native and glycated BLG was continuous, verifying intactness of Caco‐2 monolayer and preserved tight junctions during experiment.

### 3.3 Glycation of BLG Increased Uptake by BMDCs

**Figure 3:**
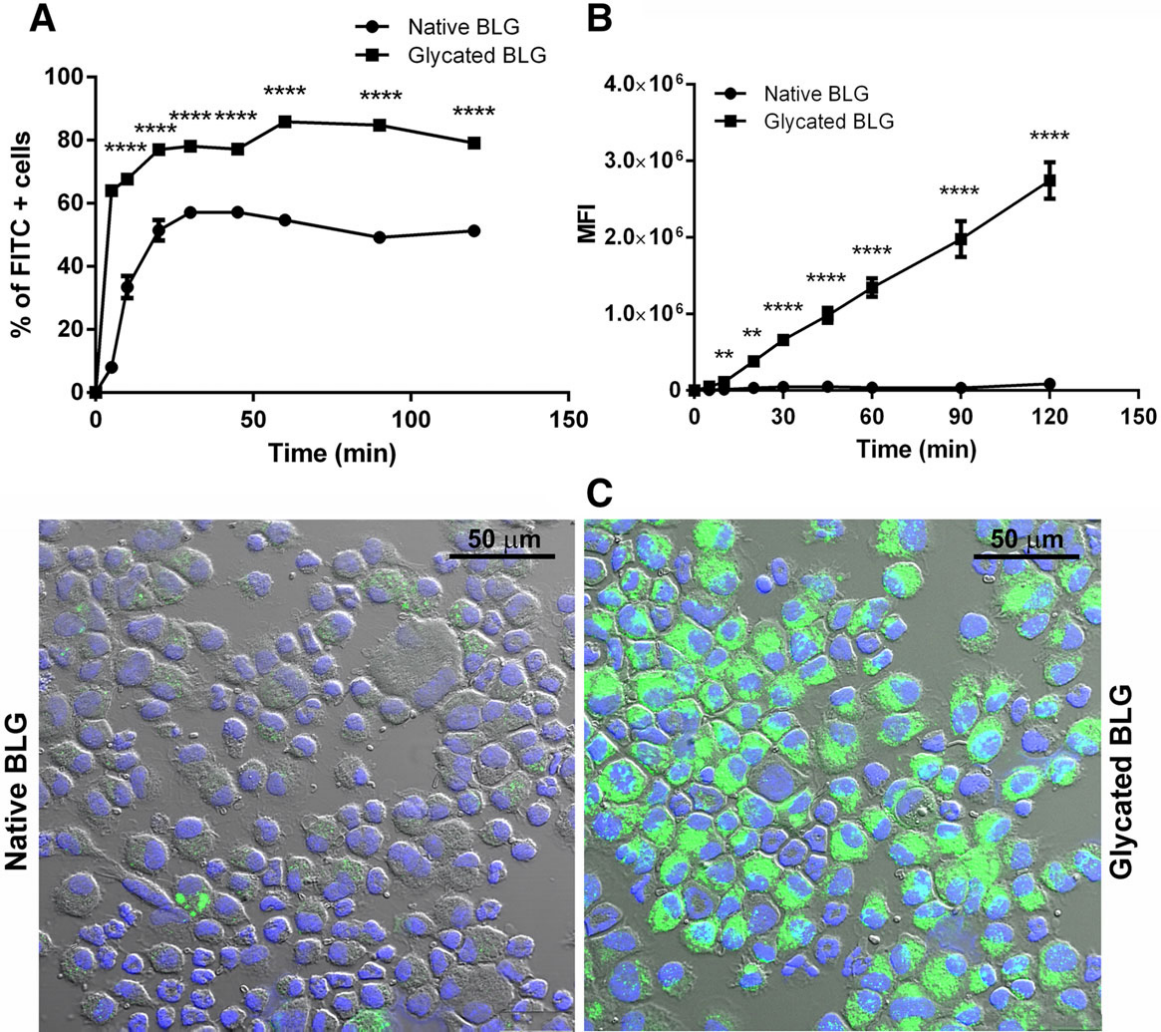
BMDCs uptake of FITC‐labeled native and glycated BLG followed in time. BMDCs were incubated with 10 μg mL^−1^ FITC‐labeled native and glycated BLG for 120 min and the BMDC uptake was analyzed by means of flow cytometry. Data are expressed as A) percent of FITC‐positive BMDCs and B) MFI. ** and **** represent significance at *p* < 0.01 and 0.0001, respectively. C) Confocal micrographs of BMDC incubated with FITC‐labeled native and glycated BLG for 30 min.

Further, we investigated whether glycation through MR influences interactions of BLG with BMDCs. FITC‐labeled native and glycated BLG were incubated with BMDCs, and BLG uptake was followed in time by flow cytometry. Uptake of both BLG forms, measured as percent of FITC‐positive cells (**Figure** 3A) was time dependent and reached plateau after 30 min, while the MFI (Figure 3B) continuously increased up to 120 min. Glycated BLG was taken up more efficiently reaching 85% of FITC‐positive BMDCs compared to 55% when incubated with native BLG. Significantly increased uptake of glycated BLG was also observed when MFI was measured.

Confocal micrographs were in agreement with the findings obtained by flow cytometry, BMDCs displayed a higher fluorescent signal upon incubation with glycated BLG (Figure 3C).

### 3.4 Glycated BLG Entered BMDCs via Receptor‐Mediated Endocytosis, Involving Scavenger Receptors

**Figure 4:**
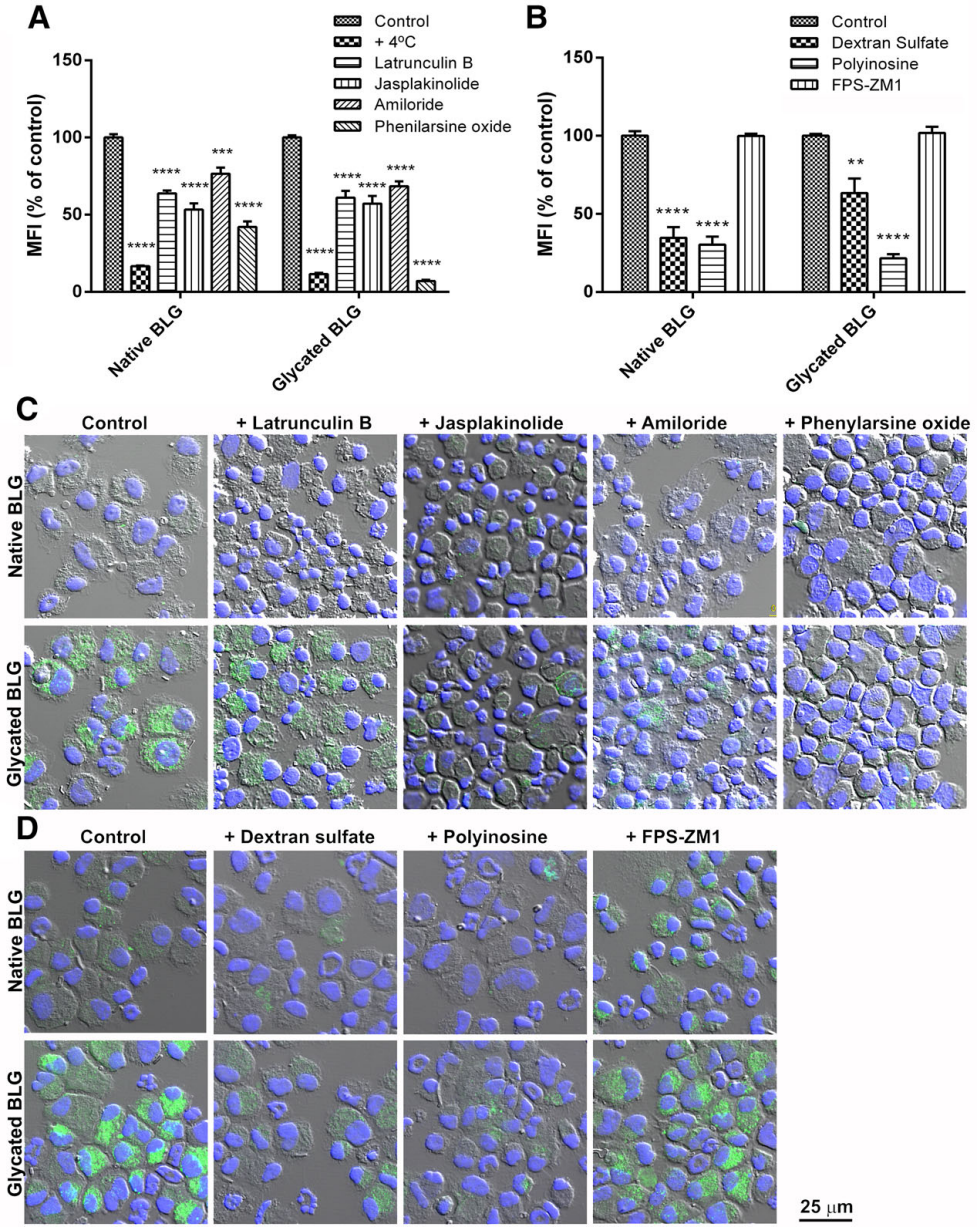
Mechanistic studies of uptake of FITC‐labeled native and glycated BLG in the presence of different inhibitors. BMDCs were pre‐incubated for 30 min with A) mechanism‐specific inhibitors, namely: latrunculin B—inhibitor of actin polymerization; jasplackinolide—inhibitor of phagocytosis; amiloride—macropinocytosis inhibitor; phenylarsine oxide—inhibitor of receptor‐mediated endocytosis, or with B) receptor‐specific inhibitors: dextran sulfate and polyinosine—SRs inhibitors; FPS‐ZM1—RAGE inhibitor. Subsequently, cells were loaded with 10 μg mL^−1^ FITC‐labeled native and glycated BLG. BMDCs uptake was analyzed by flow cytometry, and data are represented as percent of MFI of non‐inhibited uptake as mean ± SEM (*n* = 6) and analyzed by Student's *t*‐tests. **, ***, and **** represent significance at *p* < 0.01, 0.001, and 0.0001, respectively. Mechanistic studies in the presence of C) mechanism‐specific inhibitors or D) receptor‐specific inhibitors were additionally followed by confocal microscopy.

To understand the process of uptake by BMDCs of native and glycated BLG, we inhibited several possible uptake routes. First, BMDCs were incubated at 4 °C to stop all active cellular processes. This led to almost complete reduction of native and glycated BLG uptake (**Figure** 4A), indicating that the uptake was active and by an energy dependent process. Second, pretreatment with latrunculin B and jasplakinolide, which are known to interfere with actin polymerization and therefore inhibit phagocytosis, reduced uptake of both BLG forms (Figure 4A,C). This suggested that actin and phagocytosis were involved in the uptake process. Third, amiloride, an inhibitor of macropinocytosis, also decreased uptake of both BLG forms (Figure 4A,C). Final, pretreatment of BMDCs with phenylarsine oxide, an inhibitor of the internalization of cell surface receptors, resulted in a strong inhibition of the endocytosis of native BLG, while almost completely abrogated uptake of glycated BLG (Figure 4A,C). Taken together, inhibition studies showed that native and glycated BLG entered BMDCs in actin‐dependent process and phagocytosis, by constitutive macropinocytosis and by receptor‐mediated endocytosis.

Additionally, in an attempt to identify receptor(s) involved in the uptake of native and glycated BLG, inhibitors for specific endocytic receptors were used. Both dextran sulfate and polyinosine, inhibitors of SRs, remarkably reduced the uptake of both BLG forms (Figure 4B,D). Treatment of BMDCs with FPS‐ZM1, a RAGE inhibitor, did not significantly affect uptake of any BLG form (Figure 4B,D). This suggests that both BLG forms bind to and being internalized via SRs at least to some extent. Redirection of glycated BLG uptake toward receptor‐mediated endocytosis could be caused by stronger recognition by SRs due to changes in physicochemical properties that followed the MR.

### 3.5 Glycated BLG Showed Higher Susceptibility to Endolysosomal Degradation Inside BMDCs

**Figure 5:**
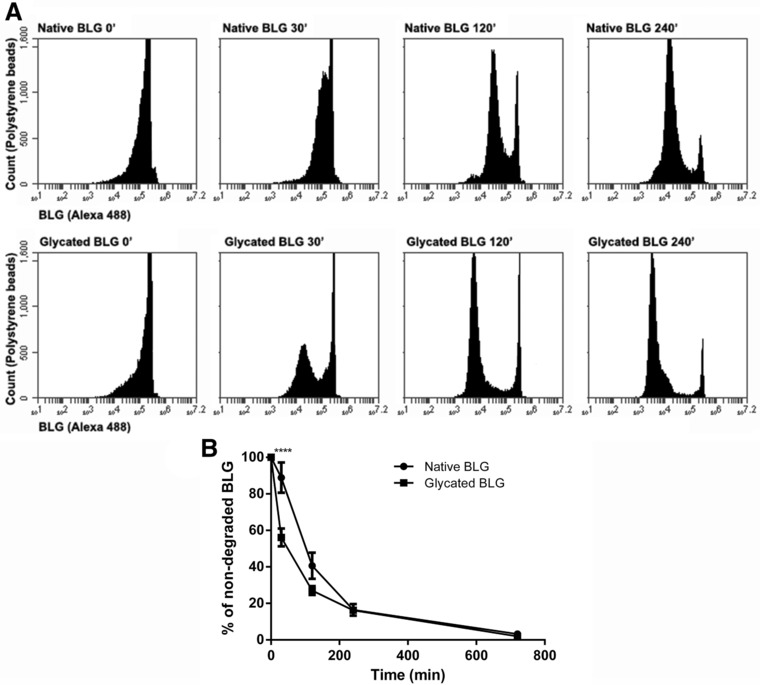
Kinetics of endolysosomal degradation of native and glycated BLG. BMDC were allowed to internalize native and glycated BLG‐coated polystyrene beads and incubated for different periods at 37 °C to allow endolysosomal degradation. After every time‐point, cells were lysed and beads carrying non‐degraded BLG were stained by Alexa 488 BLG‐specific antibodies. Polystyrene beads were analyzed by flow cytometry. A) Flow cytometry dot plot of one representative experiment. B) Percentage of non‐degraded native and glycated BLG over time is shown as mean ± SEM (*n* = 4). **** denotes significance at *p* < 0.0001 confidence level.

Efficient intracellular antigen degradation and processing is of crucial importance for antigen presentation. We therefore compared endolysosomal proteolysis of native and glycated BLG within BMDCs. BMDCs were allowed to phagocytose native and glycated BLG coupled to polystyrene beads, and endolysosomal degradation of allergens was determined by BLG‐specific antibody binding. Significant lower antibody binding to glycated BLG after first 30 min of degradation indicated that glycation influenced degradation kinetics, making glycated protein more susceptible to proteolysis especially at first time (**Figure** 5).

### 3.6 Glycated BLG Induced Lower CD4^+^ T‐Cell Cytokine Responses

**Figure 6:**
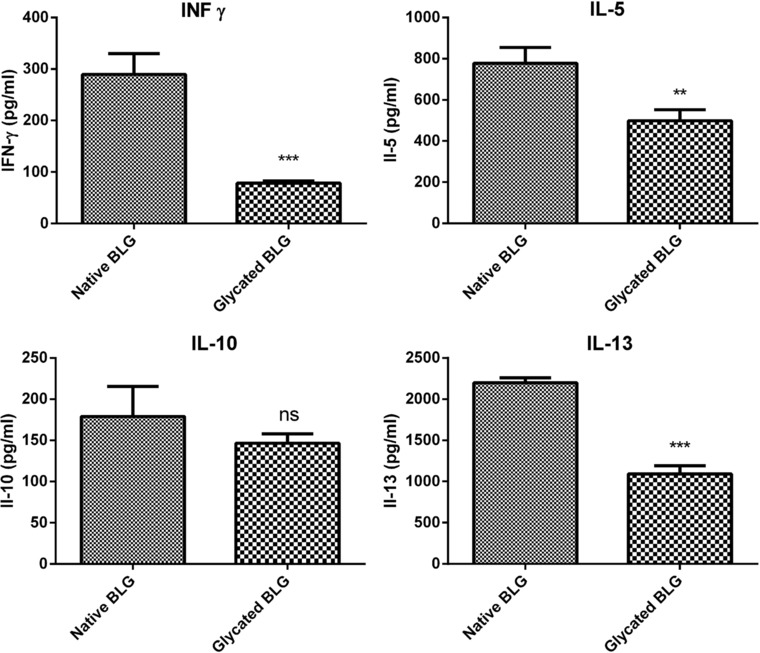
Stimulation of BLG‐specific CD4^+^ T‐cells by native or glycated BLG‐primed BMDCs. CD4^+^ T‐cells isolated from C3H/HeOuJ mice immunized with native BLG were cocultured with BMDCs primed with native or glycated BLG for 72 h. Levels of INF‐γ, IL‐5, IL‐10, and IL‐13 in coculture supernatants were measured by ELISA. The data are presented as mean ± SD of one representative experiment and analyzed by Student's *t*‐test. **, ***, and ns, represent *p* < 0.01, 0.001, and not significant, respectively.

To assess the specific T‐cell responses to glycated BLG, BLG‐specific CD4^+^ T‐cells were isolated from mice immunized to native BLG and cocultured with BMDCs primed with native or glycated BLG. After coculture, glycated BLG‐primed BMDCs induced lower production of INF‐γ, IL‐5, and IL‐13 by T‐cells, when compared with native BLG‐primed BMDCs (**Figure** 6). Both native and glycated BLG‐primed BMDCs equally induced IL‐10 production.

### 3.7 Glycation of BLG Reduced Basophil Activation

**Figure 7:**
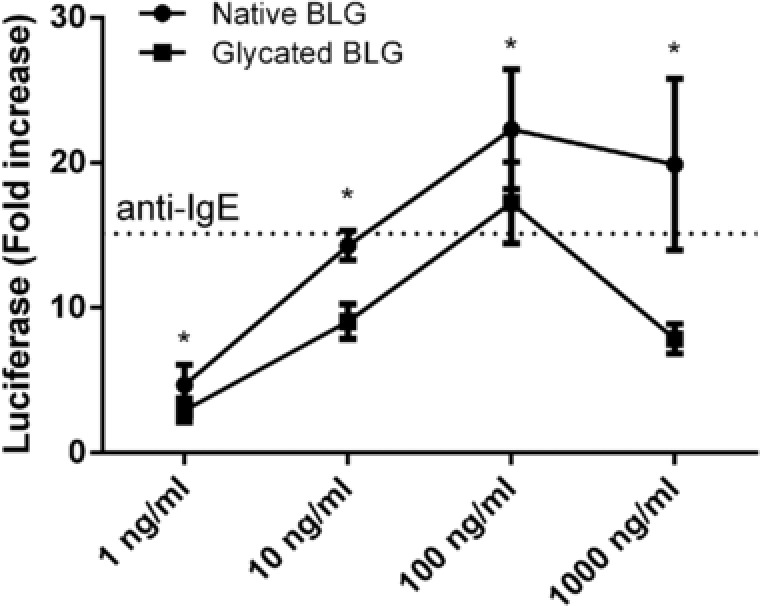
IgE crosslinking‐induced luciferase expression assay demonstrating basophil activation by native and glycated BLG. RS‐ATL8 cells were sensitized with the pool of BLG‐specific chimeric human IgE monoclonal antibodies and subsequently stimulated with 1, 10, 100, or 1000 ng mL^−1^ of native and glycated BLG. Luciferase expression level after 1 h of stimulation is shown. Dashed line shows luciferase expression after stimulation with 1 μg mL^−1^ anti‐human IgE antibodies. Data are expressed as mean ± SD (*n* = 8) and analyzed by *t*‐tests. *p < 0.05.

Finally, to gain insight into the effects of glycated BLG on allergic effector responses, a basophil cell elicitation test was performed. RS‐ATL8 cells were sensitized with oligoclonal anti‐BLG‐specific humanized IgE antibodies and subsequently native or glycated BLG was added. Glycated BLG induced significantly lower basophil activation compared to native BLG at all tested concentrations (**Figure** 7).

## 4 Discussion

Thermally processed food is highly consumed in the Western diet nowadays. Food processing can induce multiple biochemical reactions in food including MR which cause glycation of food proteins and formation of AGEs. Glycation of food allergens may have an impact on allergenicity but there is no conclusive data on how these modifications alter allergenic manifestations. The immunomodulatory effects of AGEs structures have been described in literature (reviewed in refs. 18, 30, 31, 32, 33). For instance, the effects of MR on immunogenicity and allergenicity of food allergen OVA have also been studied and have shown an elevated risk of food allergy.^15^, ^16^, ^34^ We aimed to describe how glycation reaction of the major milk allergen, BLG, influences handling and processing by epithelial and dendritic cells.

In order to exert a possible immunological effect, glycated proteins must be able to pass the intestinal epithelial barrier and to get in contact with immune system. Transport of allergens can occur transcellularly or paracellularly through intestinal epithelial cells. The main route of in vivo intestinal absorption of native BLG is through epithelial cells.^35^, ^36^ We compared the transfer of native and glycated BLG using the well‐known in vitro Caco‐2 cell monolayer system for testing intestinal barrier function. We showed that both native and glycated BLG are able to cross the Caco‐2 monolayer, although the MR drastically reduced transcytosis of BLG. This could have been caused by the physicochemical changes in BLG induced by glycation, such as partial unfolding, and formation of high molecular weight species which can make transcytosis more difficult. Reduced intestinal permeability would result in lower allergen uptake and may hence reduce activation of sensitized *lamina propria* mast cells and subsequent anaphylactic responses.^37^ Roth et al. reported that pasteurization of the BLG and α‐lactalbumin protected against an anaphylactic reaction, since uptake through epithelial cells was impaired due to aggregation.^36^ Furthermore, in our basophil activation experiments, glycation of BLG reduced its capacity to crosslink FcεRI receptors on RBL cells. In extensively glycated BLG, all residues containing free amino group (16 lysines, two arginines, and N‐terminal amino group of Leu 1) seem to be glycated,^38^, ^39^ while all of the BLG‐specific chimeric human IgE monoclonal antibodies used in this study recognize peptides containing at least 1–2 residues with possible glycation sites.^27^ Therefore, it is highly likely that extensive BLG glycation reduced mAb IgE binding by changing epitope structure, and led to reduced basophil activation. Additionally, previous studies showed a masking effect of glycation with different monosaccharides on IgE binding properties of BLG.^8^ This effect of high degree of glycation on the recognition of food allergens by IgE antibodies has been well documented in literature.^8^, ^9^, ^40^ Lower allergic effector responses (as measured by ability to induce crosslinking of FcεRI receptors), together with much lower intestinal transport (as measured in transcytosis Caco‐2 experiment) suggest that glycation of BLG reduces the risk of allergic or even anaphylactic reactions.

To further characterize the down‐stream processing of glycated BLG, we examined its uptake and degradation by murine BMDCs. We found that the uptake of glycated BLG by immature BMDCs was significantly increased, compared to uptake of native BLG, at every measured time‐point. The same was observed for uptake of glycated OVA by either murine BMDCs or human myeloid DCs.^15^, ^16^, ^34^ In addition, we used pharmacological inhibitors which interfere with different internalization pathways. Phenylarsine oxide inhibited the uptake of both native and glycated BLG, indicating that receptor‐mediated endocytosis is involved in internalization process. The more pronounced inhibition of glycated BLG uptake, compared to inhibition of native BLG uptake, in the presence of phenylarsine oxide indicates a shift of glycated BLG uptake to receptor‐mediated endocytosis. To execute their role, BMDCs are equipped with a full array of specialized receptors, including pattern‐recognition receptors (PRRs) such as toll‐like receptors (TLRs), C‐type lectins (CTLs), and SRs. Literature suggests that some allergens interact with PRR and thereby stimulate innate and adaptive responses.^41^ Considering their role in recognizing glycated products, we selectively blocked SRs and RAGE and found that SRs were responsible for the uptake of both native and glycated BLG. Our findings are in accordance with previously published data that AGEs structures of glycated proteins bind to SR class A.^34^, ^42^ Interestingly, SRs ligands are polyanionic molecules^43^ and BLG is an acidic protein with pI value 5.1, which can explain its binding to SRs. Blocking of lysyl residues by sugar moieties in MR lowers pI value and makes glycated BLG even more acidic, possibly leading to higher binding affinity for SRs and consequently higher uptake by BMDCs. Another factor influencing faster uptake could be changed folding and increased aggregation that accompanied the glycation reaction. Similarly, pyrraline, an AGEs structure, when attached to OVA has been suggested to promote SR class A‐mediated allergen uptake by DCs.^34^

In spite of the much higher uptake by BMDCs through SR‐mediated endocytosis, glycated BLG showed lower CD4^+^ T‐cell stimulation. Glycated BLG‐primed BMDCs in coculture with BLG‐specific CD4^+^ T‐cells showed significantly lower production of Th1‐ (INF‐γ) and Th2‐type cytokines (IL‐5 and IL‐13) when compared with native BLG‐primed BMDCs. This indicated that there was less peptide loading of MHC class II when BMDCs were primed with glycated BLG. Previously published results, showing that glycated OVA was taken up by DCs via SR class A type I and type II‐mediated endocytosis, followed with enhanced CD4^+^ T‐cell responses,^15^ are in contrast with our findings and indicate that overall effect on T‐cell responses is carbohydrate‐ and allergen‐dependent. These findings also stress the importance of events that follow antigen internalization, that is, antigen processing and activation of downstream signaling pathways. In support of our observations, Smole et al. reported that immature monocyte‐derived DCs were able to internalize potent allergen Bet v 1 and its structural homolog, but weak allergen, Api g 1 with the similar kinetics, and furthermore, there was no difference in the uptake by DCs from healthy or allergic donors.^44^ We showed increased susceptibility of glycated BLG to endolysosomal proteolysis. Glycation of BLG increased content of random structures (as shown by CD spectroscopy), reducing its fold stability and making polypeptide backbone more accessible to endolysosomal proteases. Intracellular antigen degradation in antigen‐presenting cells determines the antigen fate.^45^ Antigens highly susceptible to endolysosomal proteases are known to possess weak capacity of T‐cell priming.^46^ This could explain the observed decreased T‐cell stimulation by glycated BLG.

In conclusion, in this study, we found that BLG glycated in MR showed reduced transepithelial transport, but higher uptake by BMDC. It appeared that both native and glycated BLG can be endocytosed by BMDCs via SRs, but MR made BLG a better ligand for SRs possibly through lowering pI value and aggregates formation and thus increased its uptake. Glycated BLG was more prone to endolysosomal degradation and showed lower potential to induce cytokine production in coculture of BMDCs with BLG‐specific CD4^+^ T‐cells. Glycated BLG also induced lower effector response, that is, reduced basophil activation. Altogether, our results showed that glycation of BLG influences its interactions with cells involved in the (allergic) immune response, and also that glycated BLG could be less potent in the induction of Th2 type responses in a host primarily sensitized to native BLG. Interestingly, up to now, literature data about sensitizing potential of glycated BLG are still missing. Future studies are needed to elucidate the importance of MR on allergenicity and immunogenicity of BLG in vivo. The present study contributes to the knowledge regarding the effects of food processing on food allergies and it is of great importance for considerations of pros and cons of food processing.

## Conflict of Interest

The authors declare no conflict of interest.

## Supporting information

Supplementary material
Figure S1. Protein profile of heated and glycated BLG on 12% SDS‐PAGE gel under (A) non‐reducing and (B) reducing conditions. Protein bands were stained with CBB.
Figure S2. (A) CD spectra of native and glycated BLG in far‐UV spectral range. (B) Percentages of the secondary structures were estimated by CONTIN algorithm available in CDPro package based on SP29 reference set. Secondary structure fractions were compared by Student's t‐test. * and ** represent significance at p<0.05 and 0.01 confidence level, respectively.
