# Supplementary material for: Glycation of the Major Milk Allergen β‐Lactoglobulin Changes Its Allergenicity by Alterations in Cellular Uptake and Degradation
Source: Mol Nutr Food Res. 2018 Jul 29;62(17):1800341. doi: 10.1002/mnfr.201800341 (PMC6174979; doi:10.1002/mnfr.201800341)
Supplement: Supplementary file 1 — Supplementary material Figure S1. Protein profile of heated and glycated BLG on 12% SDS‐PAGE gel under (A) non‐reducing and (B) reducing conditions. Protein bands were stained with CBB. Figure S2. (A) CD spectra of native and glycated BLG in far‐UV spectral range. (B) Percentages of the secondary structures were estimated by CONTIN algorithm available in CDPro package based on SP29 reference set. Secondary structure fractions were compared by Student's t‐test. * and ** represent significance at p<0.05 and 0.01 confidence level, respectively. [file MNFR-62-na-s001.docx]

**Supplementary material**

**Glycation of the major milk allergen β-lactoglobulin changes its allergenicity by alterations in cellular uptake and degradation.**

Marija Perusko^1^, Manon van Roest^2^, Dragana Stanic-Vucinic^3^, Peter J. Simons^4^, Raymond Pieters^2^, Tanja Cirkovic Velickovic^3,5,6^, and Joost J. Smit^*2^

*^1^Faculty of Chemistry - Innovation Center d.o.o., Belgrade, Serbia; ^2^Institute for Risk Assessment Sciences, Immunotoxicology, Utrecht University, Utrecht, The Netherlands; ^3^Center of Excellence for Molecular Food Sciences & Department of Biochemistry, University of Belgrade – Faculty of Chemistry, Belgrade, Serbia; ^4^Bioceros B.V., Utrecht, The Netherlands; ^5^Faculty of Bioscience Engineering, Ghent University, Ghent, Belgium; ^6^Ghent University Global Campus, Yeonsu-Gu, Incheon, South Korea*

**METHODS**

**Isolation of BLG from cow’s milk**

Cow’s milk was centrifuged at 4000 x g for 30 min at 4 °C to obtain skimmed milk. Casein was precipitated by lowering pH to 4.6 and removed by centrifugation at 4000 x g for 30 min. Resulting whey was defatted by tetrachloroethylene extraction (2:1 v/v) and dialysed against 20 mM Tris buffer pH 7.5. DEAE Sephadex A-50 (GE Healthcare, Uppsala, Sweden) was equilibrated with 20 mM Tris pH 7.5 and adsorbed whey proteins were eluted by a salt step gradient ranging from 40 mM to 280 mM NaCl. Fractions containing purified BLG were dialysed against 50 mM phosphate buffer pH 8.0 and concentrated. Purity of the obtained BLG preparation was estimated to be >95% by SDS-PAGE. Protein concentration was determined by bicinhoninic acid (BCA) assay (Pierce, Amsterdam, The Netherlands).

**Caco-2 cell culture**

The human colon cancer cell line, Caco-2, was obtained from American Type Culture Collection (Rockville, MD, USA). Cells were cultured in DMEM (Gibco, Invitrogen, Carlsbad, CA, USA) supplemented with 1% nonessential amino acids, 20% heat-inactivated Fetal Calf Serum (FCS) and 1% of a mixture of penicillin and streptomycin (10 000 units and 10 mg/ml, respectively) at 37 °C in a humidified atmosphere of 5% CO_2_. Cells were subcultured weekly after reaching approximately 80% confluence and the culture medium was changed three times per week.

**Coupling of native and glycated BLG to polystyrene beads**

Polystyrene beads (Polysciences, Inc., Warrington, PA, USA) were pre-activated with 8% (v/v) glutaraldehyde for 4h at room temperature. Approximately 1.7 x 10^8^ beads were conjugated to native and glycated BLG (0.5 mg/ml, 0.5 ml) on a rotating wheel overnight at 4 °C. After quenching unreacted groups with 0.5 M glycine for 30 min, beads were used for endolysosomal degradation assays.

**RESULTS**

Heated BLG prepared under the same conditions as glycated BLG but in the absence of glucose (10 mg/ml BLG, 50 mM sodium phosphate buffer, pH 8.0, 60 °C for 10 days) was compared to glycated BLG in terms of protein structure and aggregation. In comparison to native BLG, heated and glycated BLG underwent different structural changes. SDS-PAGE analysis under non-reducing conditions (Fig. S1A) showed that heated sample was polymerized, with no free monomer form. Glycated BLG was present in form of monomer, dimer and polymer. Reducing SDS-PAGE (Fig. S1B) revealed heated BLG in monomer, dimer, trimer and polymer form suggesting that the most of the covalent bonds were disulfide bridges. Glycated BLG showed almost the same electrophoretic pattern under both reducing and non-reducing conditions suggesting formation of only non-disulfide covalent bonds.

| 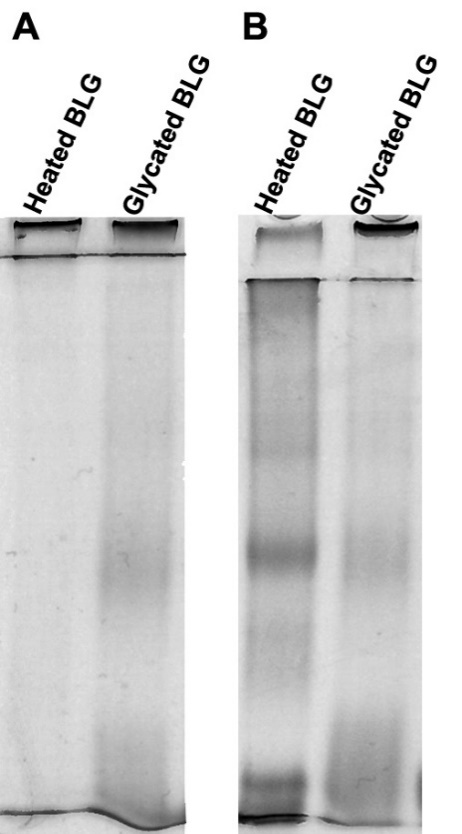 | |
| --- | --- |
| Figure S1. Protein profile of heated and glycated BLG on 12% SDS-PAGE gel under (A) non-reducing and (B) reducing conditions. Protein bands were stained with CBB. | |
| **A**   | **B**   |
| Figure S2. (A) CD spectra of native and glycated BLG in far-UV spectral range. (B) Percentages of the secondary structures were estimated by CONTIN algorithm available in CDPro package based on SP29 reference set. Secondary structure fractions were compared by Student’s t-test. * and ** represent significance at p<0.05 and 0.01 confidence level, respectively. | |

In addition, far-UV CD spectra showed significant difference in secondary structures between heated and glycated BLG (Fig. S2A). In comparison to native BLG, both heated and glycated BLG showed shift of peak minimum towards lower wavelengths, but peak of heated BLG was more profound indicating more significant loss of beta sheets. Indeed, estimation of secondary structure content using CONTIN algorithm showed significant difference between heated and glycated BLG in the content of α-helix, β-turn and β-strand (Fig. S2B). Glycated BLG was characterized with significantly (P>0.01) higher β-strand content, as well as lower α-helix and β-turn content (P>0.05), compared to heated BLG.

Taken together, the addition of sugar partially protects BLG from heat-induced denaturation, and structural changes induced by Maillard reaction are not the result of additive effect of thermal treatment and covalent binding of sugar. Maillard reaction is a complex process that results in unique protein modification rather than the simple sum of covalent attachment of sugar moieties and thermally induced structural changes. Therefore, it would have been very difficult to use heated BLG as a reference and interpret the results in comparison to the heated sample.
